# Oscillations and Filtering Networks Support Flexible Routing of Information

**DOI:** 10.1016/j.neuron.2010.06.019

**Published:** 2010-07-29

**Authors:** Thomas Akam, Dimitri M. Kullmann

**Affiliations:** 1 UCL Institute of Neurology, Queen Square, London WC1N 3BG, UK

**Keywords:** SYSNEURO, SYSBIO, SIGNALING

## Abstract

The mammalian brain exhibits profuse interregional connectivity. How information flow is rapidly and flexibly switched among connected areas remains poorly understood. Task-dependent changes in the power and interregion coherence of network oscillations suggest that such oscillations play a role in signal routing. We show that switching one of several convergent pathways from an asynchronous to an oscillatory state allows accurate selective transmission of population-coded information, which can be extracted even when other convergent pathways fire asynchronously at comparable rates. We further show that the band-pass filtering required to perform this information extraction can be implemented in a simple spiking network model with a single feed-forward interneuron layer. This constitutes a mechanism for flexible signal routing in neural circuits, which exploits sparsely synchronized network oscillations and temporal filtering by feed-forward inhibition.

**Video Abstract:**

## Highlights

► We report a mechanism for selective routing of population-coded signals ► Spatial rate codes can be reproduced as oscillation amplitude patterns ► A feed-forward interneuron layer can be tuned to act as a band-pass filter ► We show how multiple population codes can be represented in parallel by oscillations

## Introduction

Different behavioral tasks require distinct patterns of functional interaction between specialized modules in the brain. For example, when you read a book in a noisy train carriage, information from your visual stream is processed by language regions while auditory input is ignored. Then, if a conversation catches your attention, you can effortlessly switch focus, processing the auditory linguistic content that you were ignoring a moment before. A more concrete experimental example is provided by visual-attention experiments in which participants perform a perceptual discrimination task about a stimulus in one part of their visual field while ignoring simultaneously presented distracting stimuli (Brefczynski and DeYoe, 1999; Gandhi et al., 1999). These tasks require selective processing of information from the regions of the early visual system representing the task-relevant stimulus. While important in attentional processing (Berman and Colby, 2009), signal routing is likely necessary for other cognitive tasks involving distributed brain systems (Anderson et al., 2004).

Proposals for how this routing is achieved can be broadly divided into two groups: those in which asynchronous rate-coded signals are routed by dedicated switching circuits (Anderson and Van Essen, 1987; Vogels and Abbott, 2009; Zylberberg et al., 2010), and those in which changes in patterns of synchronized network activity play a key role in modulating signal flow (Crick and Koch, 1990; Salinas and Sejnowski, 2001; Fries, 2005). Consistent with the latter “oscillatory gating” hypothesis are experimental data showing task-dependent transient increases in oscillatory synchronization (Fries et al., 2001; Bauer et al., 2006; Taylor et al., 2005; Bichot et al., 2005) and frequency-selective increases in oscillatory coherence between regions during tasks thought to require their cooperation (Gregoriou et al., 2009; Siegel et al., 2008; Montgomery and Buzsáki, 2007; Popescu et al., 2009; Pesaran et al., 2008; Lee, 2003; Doesburg et al., 2008; N. Dotson et al., 2009, Soc. Neurosci., abstract).

A possible way to selectively and accurately route a target signal to a receiving population in the presence of multiple distracting inputs was recently demonstrated using a “detailed balance” nonoscillatory gating mechanism (Vogels and Abbott, 2009). Comparable capability has not so far been demonstrated using oscillatory mechanisms. Furthermore, it remains unclear how synchrony changes in population codes can mediate signal gating, what operations downstream regions must perform on their inputs to achieve gating, and how these operations can be implemented by neural network dynamics.

**Figure 1:**
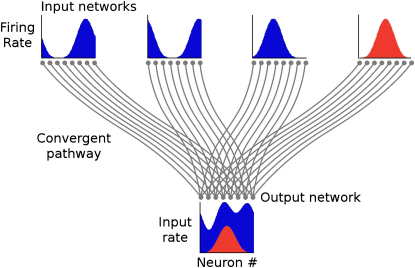
Population-Coded Information and Oscillations Input networks represent independent variables as population codes with bell-shaped firing-rate tuning curves with respect to stimulus orientation. If one input network switches from an asynchronous state (blue) to an oscillating state (red), how much more information is available to the output network about the variable that it encodes?

We have addressed these questions using a feed-forward model in which independent stimuli are represented as population codes in separate networks of spiking neurons. These populations converge to form the input to a single output network (Figure 1). When activity in all input networks is asynchronous, little information is available to the output network about the stimulus represented in any one of them, because each contributes a small fraction of the total spike input. However, we show that when one of the input networks is switched from an asynchronous to an oscillatory state, accurate information about the stimulus represented by this network becomes available to the output network. This is because in the oscillating network, the spatial pattern of firing rates is reproduced in the spatial pattern of firing-rate oscillation amplitude, thus providing a parallel channel for information transmission that is minimally affected by asynchronous distracting inputs. We further show that the filtering necessary to read out such information can be achieved by a simple and biologically plausible network of excitatory and inhibitory neurons exploiting a novel network-level resonance phenomenon. The resulting signal-gating mechanism allows accurate and selective propagation of a target signal in the presence of multiple distracting inputs. Switching among inputs can be achieved within a few cycles. Finally, we show that, when multiple input networks are oscillating in different frequency bands, filtering at the appropriate frequency can be used to pull out individual signals from the combined input, a form of frequency-division multiplexing for neural codes.

## Results

We start with a model consisting of four input networks, each of which represents a separate population-coded stimulus. These converge to form the input to a single output network. The task required of the model is to act as a switch, selecting the signal encoded in any one input network (the “sender” network), to be routed to the output network, while ignoring the signals encoded in the other input networks (“distractors”).

### Input Networks

Each input network was represented by 8000 excitatory principal cells and 2000 inhibitory interneurons, described as exponential integrate-and-fire models, mutually and reciprocally interconnected with conductance-based synapses and random connectivity. Spiking activity was induced through tonic depolarization, supplemented by a spatially patterned external Poisson spike input, used to impart stimulus tuning to the neurons as described below.

Input networks were independently switched from asynchronous to oscillatory dynamics by increasing the strength of local excitatory synapses onto interneurons, while reducing the strength of external synaptic drive to the interneurons to maintain the average firing rate of both populations (average firing rates in the asynchronous state: principal cells 4.98 Hz, interneurons 13.64 Hz; in the oscillating state: principal cells 5.34 Hz, interneurons 14.62 Hz). This switch could be equally achieved by changing other parameters such as synaptic time courses (Buehlmann and Deco, 2008) or external noise (Brunel and Hansel, 2006), or by periodic external input. The precise method does not qualitatively affect the rest of this study.

**Figure 2:**
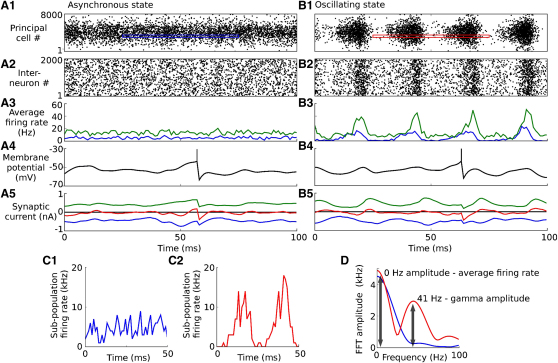
Sender Network Activity (A and B) Spike raster showing 100 ms of activity in the sender network in the asynchronous (A) and oscillating (B) states. (A1 and B1) principal cell spike raster. (A2 and B2) Interneuron spike raster. (A3 and B3) Average firing rate for the principal cells (blue) and interneurons (green). (A4 and B4) Membrane potential of sample principal cell. (A5 and B5) Synaptic currents in sample principal cell (excitatory: blue; inhibitory: green; net current: red). (C) Average firing rate of a subpopulation of 400 principal cells over a 50 ms window in the asynchronous (C1) and oscillatory (C2) states. Activity was sampled from the areas indicated by colored outlines in the spike rasters (A1 and B1). (D) Fourier transforms of the subpopulation firing rates shown in (C). Arrows illustrate the 0 Hz (average firing rate) and 41 Hz (gamma amplitude) measurements used in later analysis.

In the asynchronous state, network activity generated large excitatory and inhibitory synaptic currents in each cell, which combined to produce a small net current. Stochastic fluctuations in these currents gave rise to irregular spiking activity (Figure 2A). These features are characteristic of the balanced regime (van Vreeswijk and Sompolinsky, 1996) observed in cortical networks during up states in vivo (Haider et al., 2006; Destexhe et al., 2003) and in vitro (Shu et al., 2003).

In the oscillatory state, the instantaneous firing probabilities of both principal and inhibitory cell populations fluctuated strongly at approximately 41 Hz while individual neurons fired irregularly at lower rates (Figures 2B–2D). Average Fano factors for neurons in the oscillating state were close to 1 (0.96 for a 100 ms bin). These sparsely synchronized dynamics (Brunel and Hakim, 2008) are consistent with in vivo (Fries et al., 2001; Bragin et al., 1995; Logothetis et al., 2001; Hasenstaub et al., 2005; Csicsvari et al., 1998) and in vitro (Hájos et al., 2004) studies that show irregular activity of individual units but oscillatory activity at the network level. Excitatory and inhibitory synaptic currents oscillated approximately in phase, again consistent with recent in vivo and in vitro measurements during gamma band network oscillations (Atallah and Scanziani, 2009; Oren et al., 2006).

**Figure 3:**
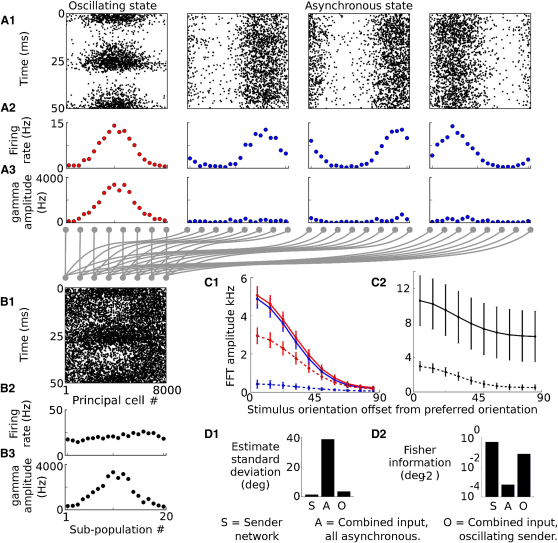
Stimulus Representation in Network Activity (A) Activity in one oscillating and three asynchronous sender networks during 50 ms. (A1) Spike raster. (A2) Average principal cell firing rate for 20 subpopulations, each of 400 neurons. (A3) Spatial pattern of gamma amplitude (population firing-rate oscillation amplitude at 41 Hz) across the 20 subpopulations. (B) Postsynaptic input obtained by summing activity in the four sender networks. (C) Average 0 Hz amplitude (solid line) and gamma amplitude (dashed line) as function of stimulus orientation for (C1) presynaptic activity in oscillating (red) and asynchronous (blue) networks and (C2) summed postsynaptic input. Error bars represent standard deviation. For the combined input, both gamma amplitude and firing rate (0 Hz amplitude) show stimulus tuning, but the gamma amplitude is far less variable because of the small contribution of distracting inputs. (D) Accuracy of stimulus representation as measured by the orientation estimate standard deviation (D1) and Fisher information (D2); estimates decoded from activity in the sender network (S) or from the combined input from sender and distractor populations when the sender network was in an asynchronous state (A) or an oscillatory state (O).

We used one-dimensional circular variables as the stimuli encoded and transmitted by the sender and distractor networks. (We make this choice for simplicity of network design and clarity of explanation, although the gating mechanism described here can be extended to more complicated stimuli.) Variables were encoded as population codes in which each principal cell had a bell-shaped firing-rate tuning curve with respect to stimulus orientation, such that they fired most strongly when the stimulus was aligned with their preferred orientation (Figure 3A2). Population codes with such tuning curves occur widely in the brain, and the one-dimensional case we consider here is analogous to orientation-selective cells in V1 or head-direction cells in the postsubiculum. This tuning was implemented by imposing a spatial pattern on the external input exciting the principal cells. Each principal cell was assigned a preferred stimulus orientation, and the external Poisson rate to each cell was determined by a cosine function of the difference between its preferred orientation and the stimulus orientation. The preferred orientation varied smoothly through 180° across the population of 8000 principal cells, so cells 1 and 8000 had a similar orientation preference, giving the network a ring topology.

We verified that the activity of the principal cells during asynchronous network activity could be decoded to produce an accurate estimate of the stimulus value using a template-matching decoding method (Deneve et al., 1999). The average spatial pattern of firing rates as a function of stimulus angle was first estimated from a training set of network activity. This spatial pattern was then used as a template for decoding by finding the stimulus estimate that minimized the mean squared error between firing rates measured over a 50 ms test period and the template of firing rates as a function of stimulus angle. To quantify decoding accuracy, we calculated the standard deviation of the stimulus estimate (standard deviation 1.40°), and the circular correlation coefficient (Fisher and Lee, 1983) between the true values of uniformly distributed stimuli and their estimates decoded from neural activity (correlation 0.998).

When the network was switched to a sparsely synchronized state, the mean firing rates of individual principal cells showed a very similar dependence on stimulus orientation as in the asynchronous state (Figures 3A2 and 3C1). Template-matching decoding continued to produce a reliable estimate of the stimulus orientation from the average firing rates over a 50 ms period of network activity (estimate standard deviation 1.22°, correlation 0.998).

We asked whether the periodic temporal structure induced by the oscillation provided new coding possibilities. One possible coding variable is the phase of firing of individual cells relative to the network oscillation, as proposed for hippocampal place cells during theta oscillations (O'Keefe and Recce, 1993). Although an analogous form of phase coding has also been proposed to occur in gamma-oscillating networks (Fries et al., 2007), we have not explored this further here. Instead, we asked whether the spatial pattern of the amplitude of oscillations in the firing rate can itself encode information about the stimulus.

Because individual neurons fired irregularly with average rates well below the population oscillation frequency, the Fourier spectrum of spike patterns for individual neurons was dominated by Poisson-like noise. The spatial pattern of oscillation amplitude became apparent only when we were looking at the firing rate of populations of principal cells with similar stimulus preference. We therefore grouped them into 20 subpopulations of 400 adjacent cells. We then evaluated the frequency spectrum of the total firing rate of each subpopulation using a short-time Fourier transform (STFT) with a 50 ms Hanning window (Figure 2D). We used two measurements from the Fourier spectrum in further analysis: the amplitude at 0 Hz (which is simply the average firing rate of the population over the window) and the amplitude at the 41 Hz oscillation frequency, which we refer to as the gamma amplitude.

We evaluated the spatial pattern of gamma amplitude across the 20 subpopulations for 50 ms periods of network activity in the asynchronous and oscillating states (Figure 3A3). In the asynchronous state, the gamma band amplitude was small, reflecting random fluctuations in network activity. However, in the oscillating state, the periodic modulation induced by the network oscillation elicited a large gamma amplitude for subpopulations whose average firing rate was high. This caused the spatial pattern of the gamma amplitude to reproduce the spatial pattern of firing rates. This spatial pattern of gamma amplitude could be decoded just like a pattern of firing rates to produce an accurate estimate of the stimulus orientation (estimate standard deviation 1.35°, correlation 0.998 using template matching).

These two different frequency components (0 and 41 Hz) of the firing rate are effectively two separate channels, each of which carries information about the stimulus orientation. Because the network generated large gamma amplitudes only in the oscillating state, when the signal from an oscillating sender network was summed with signals from asynchronous distractor networks, the gamma amplitude of the combined signal was dominated by activity in the oscillating network. Can this allow accurate estimate of the stimulus represented in the oscillating sender with minimal contamination by the distractors?

### Signal Gating among Convergent Pathways

We considered a situation in which the sender network and three distractor networks converged onto a single receiver network, such that the postsynaptic input was simply the sum of the presynaptic activity patterns (Figures 3A and 3B). When the sender network was in the same asynchronous state as the distractors, the orientation of the stimulus driving the sender network was ambiguous and could be estimated little better than chance from the combined spike input (estimate standard deviation 38.78°, correlation 0.171).

We asked whether changing the sender network to an oscillatory state increased the information available to the receiver. To do this, we analyzed the spatial pattern of gamma amplitude for the postsynaptic input using the method described above for the presynaptic activity. As before, we divided the summed input from the sender and distractor networks into 20 subpopulations, each containing afferents with similar stimulus tuning (400 from each of the four input networks). Activity in each group of afferents was pooled to produce a firing-rate signal. We analyzed this signal using the STFT to evaluate the 0 Hz and 41 Hz Fourier components for 50 ms sections of the combined input. Although the 0 Hz amplitude was uninformative (Figure 3B2), the gamma amplitude faithfully reproduced the pattern of activity in the oscillating sender network, almost as if the distracting inputs were not present (Figures 3B3 and 3C2). The stimulus driving the oscillating sender network could be accurately estimated from the gamma amplitude of the combined input (estimate standard deviation 3.53°, correlation 0.986). This improved estimation accuracy corresponded to a >100-fold increase in the lower bound on the Fisher information available to the postsynaptic network (presynaptic, 0.51°^−2^; postsynaptic, asynchronous sender, 0.00066°^−2^; postsynaptic, oscillating sender, 0.08°^−2^; Figure 3D).

### Gating Using a Spiking Network Filter

The results above demonstrate that a population-level oscillation in the sender network greatly enhances the information available to the receiver about the stimulus it encodes. Can a biologically plausible neuronal network read out this information? A reformulation of this problem is to ask whether a network of simulated neurons can reproduce the spatial activity pattern of its oscillating input in the firing rates of its output neurons, while ignoring the asynchronous component of the input it receives.

**Figure 4:**
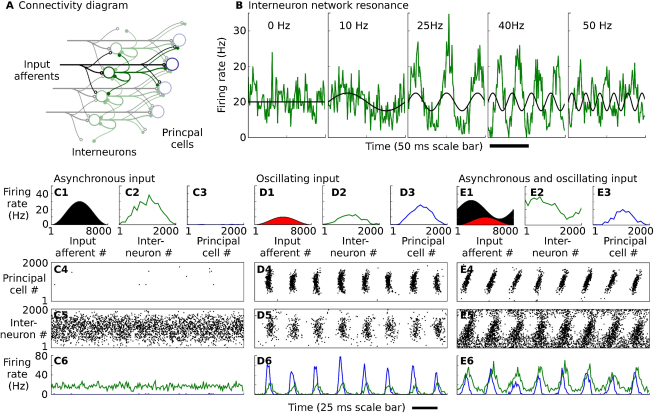
Filter Network (A) Diagram of filter network connectivity showing the input afferents (black), feed-forward interneuron layer (green), and principal cells (blue). (B) Resonance in filter network interneuron population activity. Panels show the firing rate of the interneuron population (green) driven by periodically modulated Poisson spike input (mean rate indicated in black) at a range of frequencies bracketing the network resonance frequency (see Figure S1 as well). (C–E) Filter network principal cells reproduce position of oscillating input activity, irrespective of spatial pattern of asynchronous input. Filter network activity is driven by (C) asynchronous input, (D) gamma-modulated input, (E) mixed gamma-modulated and asynchronous input. (C1, D1, E1) Spatial pattern of firing rates in afferent fibers (black: asynchronous Poisson input, Poisson input sinusoidally modulated at 40 Hz). (C2, D2, E2) Spatial pattern of the firing rate in the interneuron layer. (C3, D3, E3) Spatial pattern of the firing rate in the principal cell layer. (C4, D4, E4) Spike raster for principal cells. (C5, D5, E5) Spike raster for interneurons. (C6, D6, E6) Firing rate of principal cell (blue) and interneuron (green) populations. See Figure S2 as well.

In designing such a network, we took as our starting point the detailed balance model recently outlined by Vogels and Abbott (2009), in which an excitatory input is precisely balanced by feed-forward inhibition, such that changes in the firing rate of the input do not alter the activity of the target principal cells. We mapped the orientation-tuned excitatory inputs onto a population of 2000 principal cells, and a population of 2000 interneurons that formed a feed-forward layer, projecting to the principal cells (Figure 4A). Interneurons innervating a given principal cell received inputs from afferents with orientation tuning similar to those afferents innervating the principal cell directly (although the fine structure of the connectivity was random). This produced a pattern of feed-forward inhibitory input that balanced out the pattern of excitation in the target principal cells. Interneurons were also recurrently connected; this connectivity was local, such that only interneurons receiving input from afferents with similar orientation preference made synaptic connections with one another.

When this receiver network was driven by a bell-shaped pattern of asynchronous Poisson spike input, the pattern of input firing rates was reproduced in the interneuron layer activity and the resulting feed-forward inhibition prevented spiking in the principal cell population (Figure 4C).

To impart sensitivity to periodically modulated input patterns, we exploited a network resonance phenomenon that occurs in recurrently connected interneuron networks. Under conditions of tonic excitatory drive, interneuron networks can generate spontaneous oscillations (Whittington et al., 1995; Wang and Buzsáki, 1996; Brunel and Hakim, 1999), but these break down to an asynchronous state as the level of heterogeneity or external noise is increased (Brunel and Hansel, 2006). We found that in the asynchronous state, but near the transition to sparsely synchronized oscillation, the interneuron network acted like a damped oscillator, showing clear resonance behavior when driven by periodic input. To illustrate this resonance behavior, we drove the input afferents with a sinusoidally modulated Poisson spike input, with a range of modulation frequencies from 0 to 55 Hz (Figure 4B). The periodic modulation of the input firing rate evoked a periodic fluctuation of the firing rate of the feed-forward interneuron population. Resonance was observed both in the amplitude of the firing-rate modulation, especially prominent between 25 and 40 Hz, and in the phase of the interneuron firing-rate modulation relative to the input firing-rate modulation. The interneuron firing rate was in phase with the input at frequencies up to ∼25 Hz but lagged behind the input at higher frequencies, with approximately 90° lag for 40 Hz input modulation. This resonance phenomenon was a network effect arising from the recurrent connectivity in the interneuron layer and was not a result of subthreshold resonance in the individual interneurons (Fourcaud-Trocmé et al., 2003). In Supplemental Results and Figure S1 (available online), we show that intrinsic cellular properties combined with gap-junction connectivity can also generate resonance properties that support filtering by interneuron populations.

The 90° phase lag between the input firing-rate oscillation in the gamma band and the response of the feed-forward inhibitory population allowed the principal cells to fire in response to the oscillating input. The phase-delayed oscillations in the inhibitory firing rate disinhibited the principal cells at the phase when excitatory input was strongest. The ability to respond to spatially patterned oscillating input was tested by driving the receiver network with a bell-shaped pattern of Poisson input, sinusoidally modulated at 40 Hz (Figure 4D). This periodically modulated input activity pattern was reproduced in robust spiking activity in the principal cell layer.

For effective signal gating, the pattern of activity induced by an oscillating input should be minimally affected by converging inputs from other asynchronous networks. We tested this by driving the receiver network with the same bell-shaped pattern of periodically modulated input as in Figure 4D, but with an additional bell-shaped asynchronous Poisson input with a 3-fold larger average firing rate and a peak position rotated in orientation space from the peak of the oscillating input (Figure 4E). The spatial pattern of spiking activity in the receiver network was very similar to that induced in the absence of the asynchronous input, faithfully reproducing the location of the hump of oscillating input. The filter network was sensitive to the size of the oscillating component of the input, responding in a graded manner to changes in its average firing rate (Figures S2A and S2B).

To quantify the signal-gating performance of the filtering network, we drove it with the combined input from one oscillating sender network and three asynchronous distractor networks. Spiking activity in the filter network output layer reproduced the pattern of activity of the oscillating input regardless of the spatial pattern of asynchronous input. The stimulus encoded in the oscillating input network could be accurately decoded from the spatial pattern of filter network principal cell firing rates by using template matching on 50 ms sections of activity (estimate standard deviation 4.25°, correlation 0.978). Population firing rates in the filter network oscillated strongly, but individual neurons fired highly irregularly (mean Fano factor 1.51 for 100 ms bin).

We tested the how the gating performance varied depending on the number of distracting inputs (Figure S2C). As the number of distracting inputs increased, decoding accuracy decreased both for decoding from the gamma amplitude of the combined input and for decoding from filter network firing rates. This occurred because although each individual distractor generated a small amount of noise in the gamma band, as the numbers of distractors increased, their combined contribution grew, until it eventually drowned out the signal from the oscillating sender. As with the detailed balance gating mechanism (Vogels and Abbott, 2009), high signal propagation performance requires that the number of active distracting inputs be restricted.

For simplicity the filtering network considered above included only feed-forward inhibition. We verified that the addition of feedback inhibition did not prevent the filtering network from functioning (Figures S2D–S2I).

### Switching among Input Networks

Because the sender and distractor networks considered here had the same architecture, information about the stimulus represented in any one of the different input populations could be made available to the receiving network by switching which one was in an oscillatory state: one of the distractors could thus become the sender, and vice versa.

**Figure 5:**
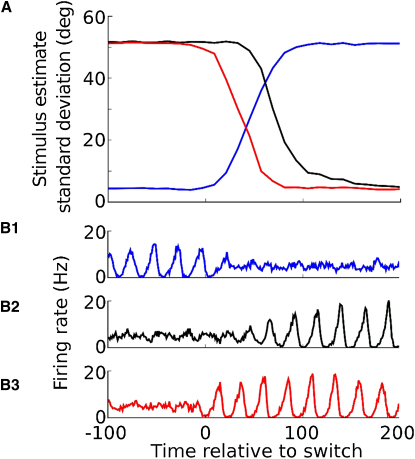
Time Course of Switching between Input Stimuli (A) Accuracy of the stimulus estimate decoded from the filter network output during the switch between input networks. (B) Firing rate in the input networks during switching. Blue traces: network switching from oscillating to asynchronous. Black traces: network switching from asynchronous to oscillating (note slow transition into oscillating state). Red traces: network rapidly switched from asynchronous to oscillating by giving the interneuron population a brief kick at the time of transition.

We tested how rapidly the filter network responded to this switching. After such a transition, it took ∼150 ms (∼6 oscillatory cycles) for the stimulus estimate decoded from the filter network to reach steady-state accuracy (Figure 5A). We observed that when a presynaptic network was switched from asynchronous to oscillatory dynamics, the oscillation did not start suddenly, but rather developed slowly over a number of cycles, limiting the speed of switching (Figure 5B2). However, by delivering a small kick to the network at the time of switching (a brief pulse of excitatory input to the interneurons), a strong oscillation could be induced immediately, reaching full amplitude on the first cycle after switching (Figure 5B3). This reduced the time taken for the filter network output to reach steady-state accuracy to 75 ms (∼3 cycles; Figure 5A). The rapid response of the filtering network could allow brief episodes of network oscillation lasting only a few cycles to transmit a pulse of information down a pathway whose default state was gated off.

### Multiplexing Population Codes in the Frequency Domain

The gating mechanism we have described works because only the oscillating sender network contributes strongly to the gamma band amplitude of the combined signal. For this reason, signal gating is severely compromised if distracting inputs also oscillate in the same frequency band (Figures S3A–S3E). However, network oscillations occur in the brain over a wide range of frequencies, raising the possibility that multiple population codes originating from networks oscillating at different frequencies could be multiplexed into a single set of inputs. This could allow the receiving network to choose which of several different inputs to respond to, by choosing which frequency band to filter from the combined input. Indeed, recent evidence from the hippocampus lends support to this hypothesis (Colgin et al., 2009). Just as frequency division multiplexing in electronic communication networks allows multiple independent signals to share the electromagnetic spectrum or optical fiber bandwidth, multiplexing may facilitate efficient use of white matter tracts in the brain.

**Figure 6:**
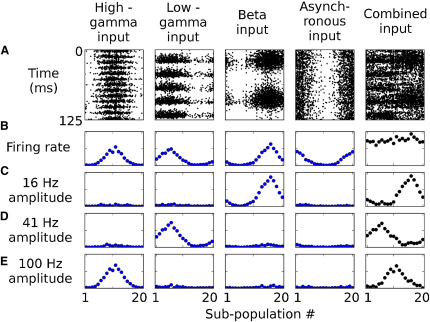
Multiplexing Multiple Signals in the Frequency Domain Four input networks, one asynchronous, one oscillating in the high gamma band, one in the low gamma band, and one in the beta band, converge to produce a combined pattern of input activity. Reading out the pattern of amplitude of the combined input at the appropriate frequency recovers the spatial pattern of activity in any one of the oscillating networks. (A) Spike rasters. (B) Spatial patterns of the firing rate. (C–E) Spatial pattern of the firing-rate oscillation amplitude in beta (C), low gamma (D), and high gamma (E) frequency bands. See Figure S3 as well.

To explore the possibility for multiplexing in our model, we replaced two of the asynchronous distracting networks with modified versions of the sender network, one of which oscillated at a high gamma frequency (∼100 Hz) and the other in the beta frequency band (∼16 Hz). The combined signal therefore contained an asynchronous, a low gamma, a high gamma, and a beta frequency component, each of which encoded a different stimulus in its spatial pattern of activity (Figure 6). The spatial pattern of activity in each of the oscillating inputs could be accurately read out from the combined signal by evaluating the spatial pattern of firing-rate oscillation amplitude at the appropriate frequency. The estimate standard deviations decoded from 125 ms sections of the combined input activity for the beta frequency input was 1.93°; for the low gamma input, 2.19°; and for the high gamma input, 1.72°. (A longer window—125 ms as opposed to the 50 ms window used for Figure 3—was used both to capture the longer period of the beta frequency oscillation and to reduce broadening of Fourier spectral peaks due to the time-frequency uncertainty principle.)

We drove the filter network with this combined input to test whether it could extract the low gamma input in the presence of distractors oscillating in other frequency bands. The stimulus encoded in the low gamma input network could be accurately decoded from the spatial pattern of filter network firing rates evaluated over 125 ms sections (estimate standard deviation 4.60°, correlation 0.976). The high correlation between the input stimulus and the estimate decoded from the filter network output shows that the filter network is able to perform the band-pass filtering needed to extract one of the three multiplexed signals. However, the accuracy was lower than that achieved by decoding from the gamma amplitude directly, suggesting that a filter network with a narrower pass-band could achieve higher signal propagation accuracy.

The filter network as outlined above was designed to extract information encoded in the low gamma band (approximately 40 Hz). However, the principles of its operation are not restricted to this frequency. The main determinants of its frequency selectivity are the resonance frequency of the feed-forward interneuron population and the synaptic time courses of excitatory and inhibitory input received by the principal cells. The former is itself strongly influenced by the time courses of synapses, although as shown in the Supplemental Information, intrinsic neuronal properties such as the afterhyperpolarization (AHP) also potentially play a major role (Figure S1). The kinetics of the conductances underlying these phenomena varies extensively across different types of interneurons, suggesting that different microcircuits may support temporal filtering operations over a wide frequency range. Furthermore, gap-junction coupling between interneurons of the same class may promote precisely the resonant network dynamics required for filtering (Bennett and Zukin, 2004; Hestrin and Galarreta, 2005). To illustrate filtering in a different frequency band, we implemented a version of the filtering network with synaptic kinetics based on the late spiking interneurons of layer 1 (Chu et al., 2003). Because of the slow time course of these conductances, the pass-band of this filtering network was tuned to a frequency of ∼10 Hz (Figures S3F–S3H).

Modulation of the amplitude of a high-frequency oscillation by the phase of a low-frequency oscillation has been observed in several brain regions (Roopun et al., 2008). We found that this arrangement reproduced the spatial pattern of firing rates in the spatial pattern of amplitude at both oscillation frequencies (Figures S3I–S3N). This suggests a possible role for these dynamics in multiplexing a stimulus into two frequency bands simultaneously.

### Time-Varying Stimuli

**Figure 7:**
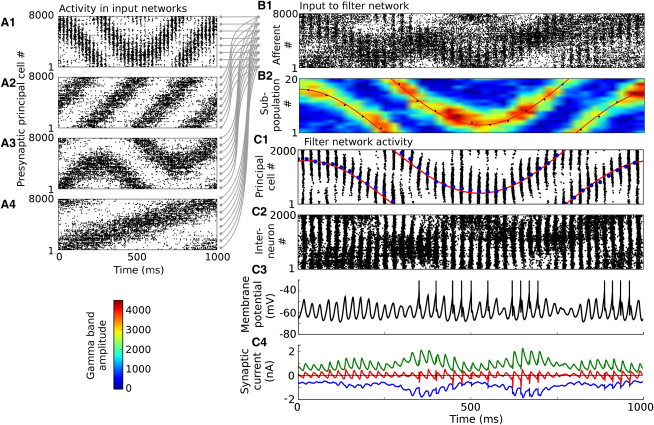
Gating Time-Varying Stimuli (A) Spike rasters of principal cells in four input networks, each encoding a different time-varying stimulus. The network generating the raster (A1) is in the oscillating state, while the other three networks are in the asynchronous state. (B) Combined input obtained by summing activity in the input networks: (B1) spike raster, (B2) spatiotemporal pattern of gamma band amplitude, together with the stimulus driving oscillating network (red line) and the decoded stimulus estimate (black dots). (C) Activity in the filtering network when driven by convergent input: (C1) principal cell spike raster (black), stimulus driving oscillating network (red), stimulus estimate decoded from pyramidal cell activity (blue), (C2) interneuron spike raster, (C3) membrane potential of a sample principal cell, (C4) synaptic currents in a sample principal cell (inhibitory: green; excitatory: blue; net current: red).

We have hitherto considered only stationary stimuli. Can the encoding scheme and the filtering network function with time-varying stimuli? We tested this by summing the activity in one oscillating sender and three asynchronous distractor networks representing stimuli that varied independently over time (Figure 7). We analyzed the spatiotemporal pattern of gamma amplitude of the combined input using a 50 ms Hanning window as before, but we moved the window along the input activity in 25 ms steps. The spatiotemporal pattern of gamma amplitude reproduced the activity in the oscillating sender network and was decoded to produce an accurate estimate of the time-varying stimulus driving the oscillating network (Figure 7B2).

We drove the input afferents of the filtering network with this convergent spike activity (Figure 7C). This induced an oscillating bump of spiking activity in the principal cells of the filtering network that followed activity in the oscillating input network. The firing rates of the principal cells in the receiver network were decoded to produce an estimator that accurately tracked the orientation of the stimulus driving the oscillating sender network (Figure 7C1). We thus conclude that, for stimuli that vary relatively slowly relative to the oscillation frequency, the mechanism described here is indeed able to route signals with high accuracy.

## Discussion

We have described a novel mechanism by which changes in the dynamical state of neural networks can turn on and off functional connectivity between anatomically connected regions.

The mechanism exploits two principles: first, in a sparsely synchronized oscillating network, a spatial pattern of firing rates is reproduced as a spatial pattern of firing-rate oscillation amplitude. This can be decoded like a conventional population code to recover the value of the encoded stimulus. Crucially, asynchronous networks or networks oscillating in different frequency bands contribute only very weakly to this amplitude pattern. Second, the pattern of oscillation amplitude at a given frequency can be read out (converted into a pattern of firing rates) by a feed-forward interneuron layer tuned to act as a band-pass filter. Importantly, and in contrast to an alternative proposal (Fries, 2005), there is no requirement for an external pacemaker to synchronize the sender and receiver networks in the scheme described here. It is sufficient for the sender network to oscillate within the pass-band for information to be selectively routed to the receiver. Of course, gating mechanisms with and without pacemaking signals are not mutually exclusive, and each may offer distinct advantages in different applications.

The gating mechanism proposed here provides several complementary approaches to modulate functional connectivity. The activity in a sending network can be switched between an asynchronous and an oscillatory state, or the frequency of the population oscillation can be varied to control how strongly it drives the pass-bands of receiving filter networks. Alternatively, the filtering performed by a receiving network on its inputs can be modulated to read out different codes multiplexed into separate frequency bands. This could be achieved by controlling the relative amount of feed-forward inhibition provided by interneuron populations implementing different filters. Neuromodulatory inputs are an attractive candidate mechanism for controlling the dynamic state of networks involved in gating. Such inputs must have sufficient spatial and cellular selectivity to change the state of local networks, but they do not need to be targeted differentially to individual cells within a population. This is a potential advantage over the detailed balance scheme (Vogels and Abbott, 2009), which requires the control input to apply a pattern of gain modulation at the single-neuron level unless each signal pathway has its own associated interneuron population.

Several forms of cross-frequency interaction, including phase-amplitude coupling (nesting) and phase locking, have been reported in network oscillations in vivo. Although we have identified one possible coding consequence of nesting (Figures S3I–S3N), a full treatment of this subject is outside the scope of the current work (Jensen and Colgin, 2007; Roopun et al., 2008; Palva and Palva, 2007).

Our study builds on previous literature addressing both the flexible routing of neural signals and the possible role of network oscillations in gain modulation. Several authors have described models in which asynchronous signals are routed by interposing dedicated circuitry between sending and receiving regions (Anderson and Van Essen, 1987; Zylberberg et al., 2010; Olshausen et al., 1993). Using oscillatory mechanisms to turn on and off direct interregion connections could alleviate the need for this additional circuitry. Prior studies have focused on how oscillatory mechanisms could reproduce effects of attention on V4 neuronal responses (Buehlmann and Deco, 2008; Niebur et al., 1993; Zeitler et al., 2008; Mishra et al., 2006). Our focus was not to reproduce a particular experimental result but rather to build a network that performs the specific computational task of signal gating—turning on and off functional connectivity for a given fixed anatomical connectivity. Studies have explored how the input-output relationship of neuronal networks can be modulated by oscillatory synchronization (Börgers et al., 2005, 2008; Masuda, 2009; Tiesinga et al., 2004; de Almeida et al., 2009; Burchell et al., 1998). Our model demonstrates significant novel functionality by allowing selective and accurate gating of population-coded signals on the basis of their oscillatory modulation, even in the presence of multiple, overlapping, distracting inputs.

Although our model generates a very high correlation between true input stimulus values and their estimates decoded from the filter network, there is inevitably a reduction in accuracy compared with stimulus representation in the input populations. This occurs because although distracting inputs are not oscillating, they produce noise in the gamma band because of their stochastic spiking, which degrades the signal. Detailed balance (Vogels and Abbott, 2009) also suffers from signal degradation as a result of the distracting inputs, because although gated-off signals are, on average, canceled by inhibition, stochastic spiking causes fluctuations that degrade the output. This is why Vogels and Abbott report that the number of active inputs must be limited for accurate signal propagation. In both mechanisms, even with a small number of active distracting inputs, the output signal is degraded in comparison to the input, resulting in reduced accuracy for a given integration time. The information rate per neuron will therefore be reduced in pathways in which multiple distracting inputs are active and flexible signal routing is required. This may be one factor contributing to reduced psychophysical performance in discrimination tasks in the presence of distracting stimuli (Pelli and Tillman, 2008; Seidemann and Newsome, 1999).

The output of the filtering network we have described is itself oscillating, and hence the signal gating mechanism is not recursive. In other words, the output of the filtering network would need to be desynchronized before it could be used as the input to another convergent pathway employing the same gating mechanism. Possible desynchronization mechanisms include neurons with subthreshold bursting dynamics to sustain spiking over the phase when they are not receiving strong input, or low pass filtering provided either by slow synaptic conductances, such as NMDA receptors, or by intrinsic neuronal properties, such as dendritic filtering.

A key signature of the described oscillatory gating mechanism is strong, coherent oscillation between sending and receiving regions during communication. Task-dependent increases in strength and interregion coherence of network oscillations have been reported in numerous brain systems, including the visual system during attentional processing (Fries et al., 2001; Siegel et al., 2008; Buschman and Miller, 2007), the hippocampus (Colgin et al., 2009) during memory tasks (Montgomery and Buzsáki, 2007), between the amygdala and striatum during learning (Popescu et al., 2009), the frontal and parietal cortex during working memory (N. Dotson et al., 2009, Soc. Neurosci., abstract) and decision making (Pesaran et al., 2008), and within the motor system during movement tasks (Lee, 2003; Schoffelen et al., 2005). Especially consistent with our model are those studies that have shown strong task-dependent synchronization in a narrow frequency band between distinct brain regions (Popescu et al., 2009; Pesaran et al., 2008). In both these studies, the oscillatory activity was limited to a subpopulation of cells recorded in the relevant regions, and the oscillations were confined to short bursts. This dispersal of oscillatory activity suggests that without careful analysis the strength of oscillatory events may be underestimated as a result of spatial and temporal averaging.

Finally, the mechanism described here makes strong predictions for the intracellular currents during signal gating. If a network is gating “on” a subset of its inputs using the mechanism that we describe, the spike rate in principal cells will be strongly correlated with the amplitude of oscillatory fluctuation in its synaptic input. Additionally, spiking activity will be strongly correlated with a phase shift between periodic modulation of excitatory and inhibitory synaptic currents. These experimentally testable predictions are distinct from gating mechanisms, such as detailed balance (Vogels and Abbott, 2009), that do not rely on oscillatory synchronization.

## Experimental Procedures

Simulations were performed with the software NEST (Gewaltig and Diesmann, 2007), except those for Figure S1 and Figures S3F–S3H, which were performed in BRIAN (Goodman and Brette, 2009). A 0.1 ms time step was used for numerical integration. All data analysis and plotting were carried out in python with the use of the Ipython, Numpy, Scipy, and Matplotlib libraries.

### Neurons

All neurons were described as exponential integrate-and-fire models (Fourcaud-Trocmé et al., 2003), and synapses were conductance based with alpha-function time courses. The membrane potential of the exponential integrate-and-fire neuron obeys the following equation:

$$C\frac{dV}{dt}=-g_{l}\left(V-E_{l}\right)+g_{l}\Delta_{T}e^{\left(\frac{V-V_{T}}{\Delta_{T}}\right)}-g_{e}\left(V-E_{e}\right)-g_{i}\left(V-E_{i}\right)+I_{e}$$

When the membrane potential reaches the spike cutoff of 0 mV, it is reset to −65 mV.

The following parameters were the same across all neuron populations: membrane capacitance *C* = 100 pF, leak conductance *g_l_* = 10 nS, leak reversal potential *E_l_* = −60 mV, spike threshold *V_T_* = −50 ± 2 mV, slope factor *Δ_T_* = 2 mV, excitatory synaptic reversal potential *E_e_* = 0 mV, inhibitory reversal potential *E_i_* = −80 mV.

Synaptic time courses, synaptic conductances, and tonic current were different across populations:

Sender network principal cells: excitatory alpha function τ = 4 ms, inhibitory τ = 3.5 ms, tonic current *I_e_* = 30 ± 20 pA.
Sender network interneurons: excitatory τ = 4 ms, inhibitory τ = 3 ms, tonic current *I_e_* = 30 ± 80 pA.
Receiver network principal cells: excitatory τ = 4 ms, inhibitory τ = 4 ms, tonic current *I_e_* = 30 ± 20 pA.
Receiver network interneurons: excitatory τ = 4 ms, inhibitory τ = 8 ms, tonic current *I_e_* = 30 ± 20 pA.

Heterogeneity was introduced in the populations by randomly varying the spike threshold and tonic current across individual cells according to a normal distribution. These parameters are quoted as mean ± standard deviation.

### Sender and Distractor Networks

The sender and distractor networks consisted of 8000 excitatory and 2000 inhibitory neurons with random internal connectivity. Each principal cell in the network received synaptic input from randomly selected 400 principal cells and 200 interneurons. Each interneuron received synaptic input from randomly selected 400 principal cells and 100 interneurons. Each pyramidal cell received external Poisson spike input, with a rate *R_ex_* (in Hz) given by

$$R_{ex}=400+140\;\cos\left(2\left(\theta_{stim}-\theta_{pref}\right)\right)\text{,}$$

in which *θ_stim_* was the current stimulus orientation and *θ_pref_* was the neurons' preferred orientation. Each interneuron received external Poisson input with a constant rate of 400 Hz. In the asynchronous state, alpha-function peak conductivities (in nS) between external inputs (X), interneurons (I), and pyramidal cells (E) were: E-E = 0.1, E-I = 0.2, I-E = 0.6, I-I = 1.5, X-E = 1, X-I = 0.8. To switch the network into the oscillating state, the synapses from excitatory to inhibitory and from external to inhibitory were modified to E-I = 0.3, X-I = 0.4.

### Sender and Distractor Network Variants

For Figure 6, the sender network was modified by changing synaptic weights and time courses to produce two variants, one of which oscillated at ∼16 Hz and one of which oscillated at ∼100 Hz.

The following are 16 Hz beta frequency synaptic parameters:

Principal cells: excitatory alpha function τ = 12 ms, inhibitory τ = 15 ms.
Interneurons: excitatory τ = 12 ms, inhibitory τ = 15 ms.
Alpha-function peak conductances (in nS): E-E = 0.05, E-I = 0.08, I-E = 0.1, I-I = 0.2, X-E = 0.47, X-I = 0.5.

The following are 100 Hz high gamma frequency synaptic parameters:

Principal cells: excitatory alpha function τ = 4 ms, inhibitory τ = 3.5 ms.
Interneurons: excitatory τ = 4 ms, inhibitory τ = 2 ms.
Alpha-function peak conductivities (in nS): E-E = 0.1, E-I = 0.2, I-E = 0.6, I-I = 2.5, X-E = 1.55, X-I = 0.8.

For Figures S3A–S3E three variant networks were created, with parameter values between those used for the asynchronous distractor network and those used for the oscillating sender network. These spanned the transition from asynchronous to sparsely synchronized dynamics. The only parameters changed were the synaptic strengths from the external input to the inhibitory interneurons and from the principal cells to the interneurons. In order of increasingly oscillatory dynamics, the modified alpha-function peak conductances (in nS) in the three variants were as follows:

Variant one: X-I = 0.6, E-I = 0.24.
Variant two: X-I = 0.5, E-I = 0.26.
Variant three: X-I = 0.45, E-I = 0.28.

### Receiver Network

The receiver network consisted of 8000 input afferents, 2000 principal cells, and 2000 interneurons. The input afferents each had a preferred orientation, which varied smoothly across the population from 0° to 180°. Orientation tuning of interneuron and principal cell activity was inherited entirely from their connectivity to the input afferents; however, in describing the connectivity pattern, it is useful to think of each pyramidal cell and interneuron as having a predefined orientation preference that again varied across the population from 0° to 180°. For each connection type (for example, interneuron to principal cell), the average connection weight between a presynaptic cell and a postsynaptic target was defined as a Gaussian function of the separation of their orientation preferences. The Gaussian function was specified for each connection type by a width in orientation space and an average total number of afferents of that type received by each cell. The width of the connection pattern is the expected standard deviation of orientation preferences of the afferents received by a cell. For the connections between input afferents (X), principal cells (E), and interneurons (I), these connection widths were X-I = 13.5°, X-E = 13.5°, I-I = 13.5°, I-E = 17°. The average numbers of afferents received by a postsynaptic cell for each connection type were X-I = 100, X-E = 600, I-I = 200, I-E = 200. Specific connections onto each cell were generated stochastically, with the number of connections between a presynaptic and postsynaptic pair drawn from a Poisson distribution with a mean determined by the Gaussian distributions described above. The peak conductances (in nS) of the alpha function synaptic time course for each connection type were X-I = 0.3, X-E = 0.1, I-I = 0.1, I-E = 0.75.

### Filter Network Variants

Alternative implementations of the filtering network were created for Figure S1. For simplicity, we did not implement a spatially mapped version of these networks in which different cells had different orientation preferences; instead, there was a single population of 800 input afferents whose firing rate was homogeneous. These projected to a population of 400 interneurons and 400 principal cells. The interneuron population was recurrently connected and made connections onto the principal cells. The connection probability between cells in each pathway (X-E, X-I, I-E, I-I) was such that the average number of inputs received by cells of each type was the same as in the original filtering network. Except where stated otherwise below, neuronal and synaptic parameters were the same as in the original filter network.

In the first variant filter network (Figure S1C), interneurons had a spike AHP current modeled as an alpha-function conductance with τ = 10 ms, conductance 18 nS, and reversal potential −80 mV. Interneurons in this network were recurrently connected with gap junctions with a conductance of 0.04 nS and a connection probability of 0.5. The tonic current received by interneurons was 50 ± 20 pA, and the alpha function τ of I-I synapses was 3 mS.

The second filter network variant had two interneuron populations: a feed-forward (FF) population and an interneuron-selective (IS) population (green and purple, respectively, in the network diagram in Figure S1D). For both populations, neuronal parameters and connections from the input afferents were the same as for the interneurons in the original filtering network. To describe the inhibitory connectivity, we state for each pathway the alpha function τ, peak conductance, and average number of synapses of each type received by a postsynaptic cell. FF-FF: τ = 3 ms, peak conductance = 0.1 nS, n synapses = 200. FF-principal cell: τ = 4 ms, peak conductance = 0.75 nS, n synapses = 200. IS-IS: τ = 8 ms, peak conductance = 0.1 nS, n synapses = 200. IS-FF: τ = 8 ms, peak conductance = 0.1 nS, n synapses = 200.

For comparison, we also implemented a pathway with no feed-forward inhibition (Figure S1A). Each principal cell received, on average, 600 synaptic connections from input afferents with alpha function τ = 4 ms and peak conductance = 0.018 nS.

For Figures S2E and S2F, we added an additional population of feedback interneurons to the filtering network. These had the same intrinsic properties as the normal interneurons in the filtering network except for the tonic current, which was 120 ± 20 pA. Each feedback interneuron received 100 excitatory synapses from randomly selected filter network principal cells with alpha function conductance τ = 4 ms, peak conductance = 0.6 nS, and reversal potential = 0 mV. Each feedback interneuron received 100 recurrent inhibitory synapses from randomly selected feedback interneurons with alpha function conductance τ = 3 ms, peak conductance = 1.5 nS, and reversal potential = −80 mV. Each filter network principal cell received 100 inhibitory synapses from randomly selected feedback interneurons with alpha function conductance τ = 4 ms, peak conductance = 0.6 nS, and reversal potential = −80. The tonic current received by filter network principal cells was increased to 120 ± 20 pA.

For Figures S2G–S2I, we added a feedback connection from the filter network principal cells to the feed-forward interneurons. Each interneuron received 100 excitatory synapses from randomly selected filter network principal cells with alpha function conductance τ = 4 ms, reversal potential 0 mv. In Figures S2G and S2H, the peak alpha function conductance is 0.075 nS, whereas in Figure S2I it is 0.15 nS.

For Figure S3F, we implemented a filtering network with the pass band tuned to 10 Hz. The filter network was identical to that used in Figure S1B, except for synaptic time scales and strengths, which were as follows: Peak conductances: X-I 0.3 nS, X-E 0.1 nS, I-E 0.8 nS, I-I 0.1 nS. Alpha function τ: X-I 8 ms, X-E 8 ms, I-E 15 ms, I-I 30 ms.

### Simulations and Analysis

For each network configuration, we performed 200 simulations of 1 s of network activity The initial 100 ms of all simulations were discarded to prevent start-up transients contaminating the data.

To quantify the 0 Hz and gamma amplitude, as well as the decoded estimation accuracy, simulations were divided into nonoverlapping 50 ms sections. In all convergent pathway conditions, the orientations of the target and distractor stimuli were randomly drawn from uniform distributions. The spatial pattern of 0 Hz amplitude (average firing rate) and 41 Hz gamma amplitude for each section was analyzed by dividing the neurons into 20 subpopulations and pooling the activity in each group to produce a firing-rate signal (all population firing rates were discretized with a 1 ms time bin). The Fourier transform of these 20 firing-rate signals was analyzed with the use of a 50 ms Hanning window to reduce spectral leakage. The amplitude of the Fourier transforms was measured at 0 Hz, to give the population average firing rate, and at the 41 Hz oscillation frequency.

### Decoding

The 50 ms sections were divided into a training set and a test set. The spatial pattern of average firing rates and gamma amplitude across 20 subpopulations was calculated for each 50 ms section as described above. The training set was used to produce a template of the average firing rate (or gamma amplitude) *R* as a function of the orientation offset *θ* between the population's preferred orientation and the stimulus orientation. The template used was a least-squares fit of a Von Mises distribution to the average spatial pattern of firing rates (or gamma amplitudes) over the training set:

$$\text{Template function}:R(\theta )={\mathrm{Ae}}^{\left(k\;\text{cos}(2\theta )\right)}$$

This template function was then used to decode an estimate of the stimulus orientation for each section in the test set by finding the orientation estimate *θ_est_* that minimized the least-squared error between the measured firing rates (or gamma amplitudes) and the template function. We report the standard deviation of these estimates over the training set, the lower bound on the Fisher information given by the reciprocal of the variance and the circular correlation coefficient (Fisher and Lee, 1983) between the uniformly distributed target stimuli and their decoded estimates.

## Supplemental Information

Document S1. Supplemental Results and Three Figures
