# Supplementary material for: Oscillations and Filtering Networks Support Flexible Routing of Information
Source: Neuron. 2010 Jul 29;67(2-10):308–20. doi: 10.1016/j.neuron.2010.06.019 (PMC3125699; doi:10.1016/j.neuron.2010.06.019)
Supplement: Document S1. Supplemental Results and Three Figures [file mmc1.pdf]

Neuron, Volume *67*

Supplemental Information

Oscillations and Filtering Networks

Support Flexible Routing of Information

Thomas Akam and Dimitri M Kullmann

Supplemental Results

Alternative Filter Networks

The biological implementation we describe in the main body of the paper is not the only way that the dynamics required for filtering can be generated. Here we illustrate two alternative filter networks which achieve the same functionality based on the same dynamical principles but with different biological implementation.

At the algorithmic level (Marr and Poggio, 1976), the filtering network encodes the amplitude of firing rate oscillation in its input afferents at a given frequency, into the firing rate of its output afferents. At the mechanistic level, asynchronous input is canceled by feed-forward inhibition, but oscillatory input induces large amplitude phase delayed oscillations in the inhibitory firing rate due to a strong resonance in the interneuron population at a lower frequency than that of the input oscillation. This disinhibits the principal cells at the phase when excitation is strong, allowing them to fire.

In the original filter network the low frequency resonance in the inhibitory population was generated by slow recurrent synapses among the interneurons. However, the inhibition received by principal cells must decay quickly to produce periods of dis-inhibition in response to oscillating inputs. This necessitated faster kinetics at the interneuron-principal cell synapses than at interneuron-interneuron synapses. This slower inhibition onto interneurons relative to excitatory cells is atypical of many interneuron populations (Jonas et al., 2004), prompting us to explore whether other biological mechanisms could produce the required resonance in the interneuron population.

Before discussing these alternative filtering networks, we note that late spiking interneurons of cortical layer 1 exhibit considerably slower kinetics at their recurrent synapses than at their synapses onto pyramidal cells (Chu et al., 2003). These interneurons are ideally located to control integration of activity in the long-range collaterals of layer 1. The slow synaptic kinetics of these interneurons suggests that if they are acting as a filtering network, the pass-band would be situated in the alpha or beta frequency range. The anatomical location and synaptic properties of these cells, in combination with in vivo data implicating low frequency oscillations in tasks involving long-range interactions, make these cells a promising candidate implementation of the described filtering network.

In the first alternative version of the filtering network (Figure S1C), low frequency resonance in the interneuron population was induced by a combination of spike after-hyperpolarisation and gap junction connections between interneurons. In this mechanism, the gap junctions promoted synchronization of the interneuron population at a frequency that was determined by the AHP time-course. The filtering performance of the network was very similar to that of the original filtering network (Figure S1B2, C2). We expect that resonance can be conferred on neuronal population responses by other features contributing to intrinsic cellular dynamics such as sub-threshold voltage-gated conductances (Brunel et al., 2008). Gap junction coupling may promote the transfer of sub-threshold dynamics to population responses by homogenizing membrane potential trajectories across populations.

Although it is conceptually simple to have a particular filtering function implemented in a single interneuron population, it is possible that more than one interneuron type participates. In a second alternative implementation of the filtering network, the slow inhibitory synapses that generated resonance at the required frequency were expressed in a separate population of interneurons from those mediating fast feed-forward inhibition (Figure S1D). The 'slow' population made inhibitory connections both recurrently and onto the 'fast' population. The 'fast' population made synapses recurrently and onto the principal cells. Recurrent connections in the 'slow' population induced resonance at a low frequency causing large amplitude, phase-shifted oscillations in its firing rate in response to oscillating input. These firing rate oscillations were imposed on the 'fast' interneurons by the synaptic connection between the two populations.

Though differing in their precise biological implementation, due to their common dynamical mechanism, these alternative filtering networks share characteristic, experimentally testable electrophysiological features. Specifically, spike output of the principal cells is associated with large amplitude oscillation in the excitatory and inhibitory synaptic current rather than being determined by the average excitatory synaptic input. Additionally, spiking activity is associated with a phase shift between oscillation in the excitatory and inhibitory synaptic currents received by the output cells of the filtering network.

Feedback Inhibition

The filter network described in the main body of the paper contains only principal cells and a feed-forward interneuron layer. We used this greatly simplified structure to illustrate the minimal circuitry needed to implement the filtering. However, real neural circuits are more complex; feedback inhibition is ubiquitous and many interneuron populations mediate both feed-forward and feedback inhibition. We tested whether filtering could continue to function in the presence of feedback inhibition.

We first tested whether filtering continued to function in the presence of an additional population of

interneurons mediating feedback inhibition of the principal cell population. Filtering continued to function effectively in the presence of feedback inhibition (Figure S2E,F).

We then tested whether a feedback connection from the principal cells to the interneurons disrupted the filtering function. As long as the feedback connection was not too strong, filtering continued to function effectively (Figure S2G,H). As the strength of feedback connection was increased, a threshold was reached above which the principal cell interneuron loop started to oscillate spontaneously, even when driven by asynchronous input (Figure S2I). This caused principal cells to fire strongly in response to asynchronous input and hence the filtering no longer functioned. This transition to spontaneous oscillation occurred when the feed-back synapses were approximately 1/3 the strength of the feed-forward synapses (each interneuron received an equal number of feed-forward and feed-back excitatory synapses).

Figure S1 (related to Figure 4)

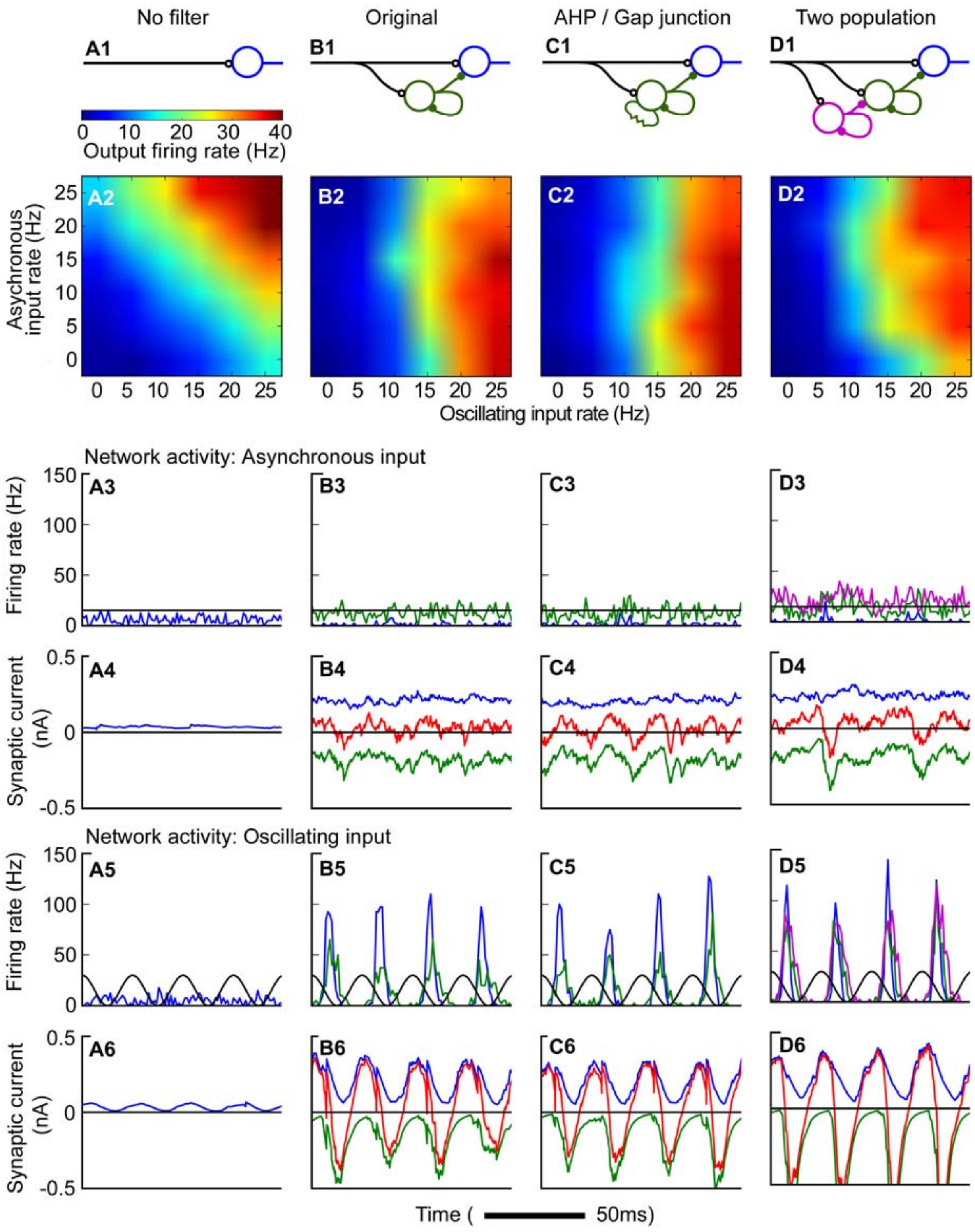

Figure S1 (related to Figure 4)

Alternative Filter Network Implementations

Two alternative filter networks were designed (**C**, **D**), which perform the same function as the original filtering network with alternative biological implementation of the resonant feed-forward inhibition. These are compared with the original filtering network (**B**) and a pathway with no feed-forward filtering (**A**). For simplicity, we show results for a filtering network in which all input afferents have the same firing rate. (**A1-D1**) Filter network diagrams showing input afferents (black), interneurons (green, purple) and principal cells (blue). The input consisted of Poisson spiking activity in the input afferents with an asynchronous and an oscillating component. The firing rate of the oscillating component was sinusoidally modulated at 40 Hz. (**A2-D2**) To illustrate each network's ability to respond differentially to the asynchronous and oscillating inputs, the firing rate of the filter network principal cells was measured as the average firing rates of the asynchronous and oscillatory components of the input were systematically varied. For all three networks incorporating resonant feed forward inhibition **(B2-D2)**, the output firing rate responded strongly to increases in the firing rate of the oscillating input component (abscissa), while changes in the asynchronous input (ordinate) had little effect. In the pathway with no filtering (**A2**) the output rate responded strongly to changes in both components of the input. (**A3-D3**) Neuronal firing rates in response to asynchronous input (traces colored as in diagrams **A1-D1**). (**A4-D4**) Synaptic current in a sample principal cell in response to asynchronous input (blue – excitatory current, green – inhibitory current, red – net current). (**A5 – D5**) Neuronal firing rates in response to oscillating input. (**A6 – D6**) Synaptic currents in a sample principal cell in response to oscillating input.

Figure S2 (related to Figure 4)

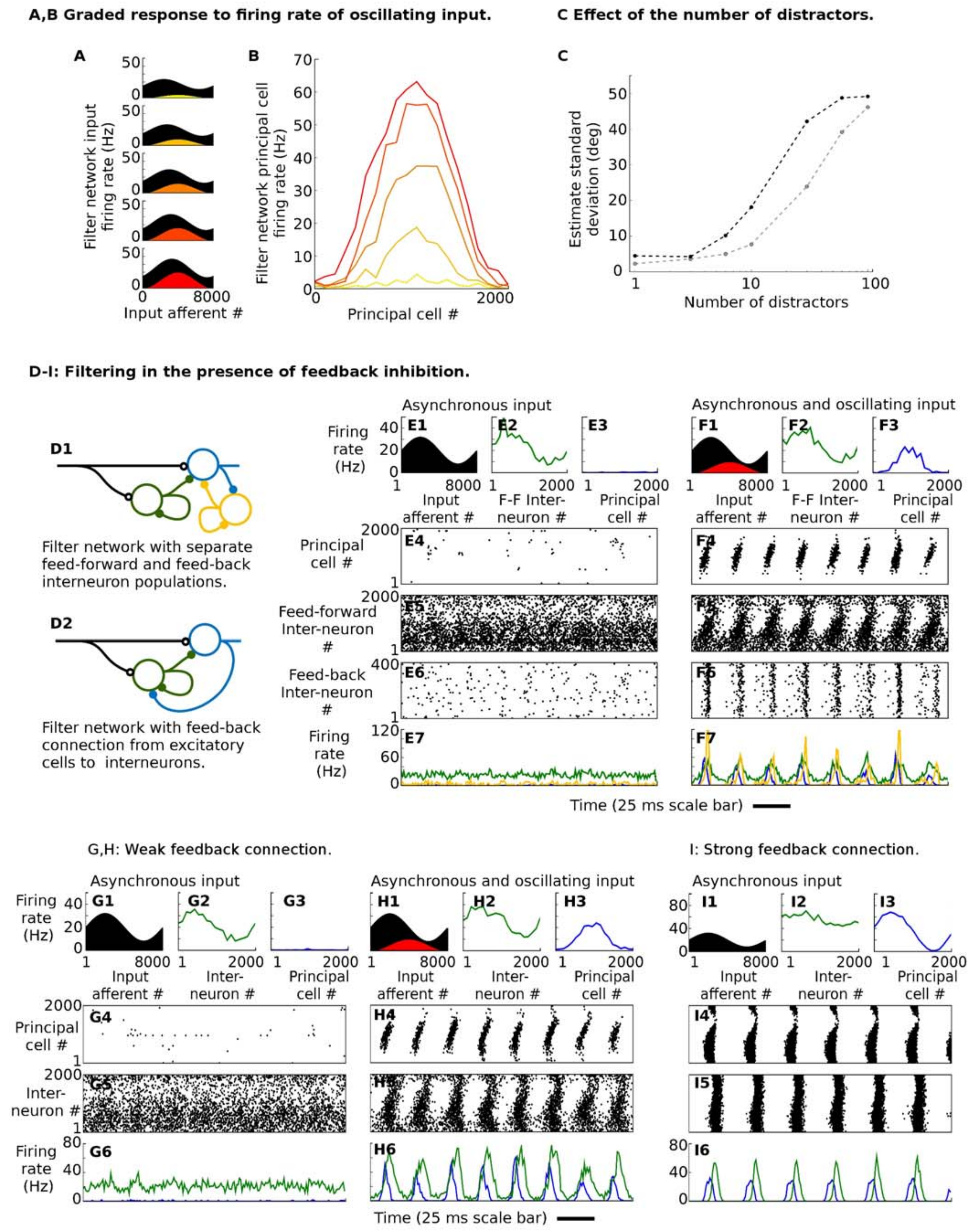

Figure S2 (related to Figure 4)

(A,B) Graded Response of Filtering Network to the Average Firing Rate of Oscillating Input

The filtering network was driven with a Poisson spike input consisting of an asynchronous and an oscillating component (sinusoidally modulated at 40 Hz). The firing rate of the asynchronous component was held fixed while the average firing rate of the oscillating component was varied (average firing rate per input afferent indicated by color, from 2 Hz (yellow) to 10 Hz (red) in 2 Hz steps). (**A**) Spatial pattern of activity in the input afferents showing the asynchronous (black) and oscillating (colored) components. (**B**) Spatial pattern of firing rate of filter network principal cells averaged over 500ms.

(C) Effect of the Number of Distractors

Traces show standard deviation of stimulus estimate as function of the number of distracting inputs, for estimates decoded from the filter network output (black trace), or the combined spike input (gray trace).

(D-I) Filtering in the Presence of Feedback Inhibition

Filter network with an additional population of feedback interneurons (**D1**), driven by (**E**) asynchronous input, (**F**) combined asynchronous and oscillating input. (**D2**) Filter network with connection from principal cells to interneurons. With a weak feedback connection, filtering continues to function effectively (**G,H**); above a threshold strength of feedback connection, the E-I loop starts to oscillate spontaneously when driven by asynchronous input (**I**). (**E1- I1**) Spatial pattern of firing rates in afferent fibers (black - asynchronous Poisson input, Poisson input sinusoidally modulated at 40 Hz). (**E2- I2**) Spatial pattern of firing rate in interneuron layer. (**E3- I3**) Spatial pattern of firing rate in principal cell layer. (**E4-6, F4-6, G4-5, H4-5, I4-5**) Spike rasters, cell type indicated by label. (**E7, F7, G6, H6, I6**) Firing rate of principal cell and interneuron populations, line color corresponds to color of populations in diagram.

Figure S3 (related to Figure 6)

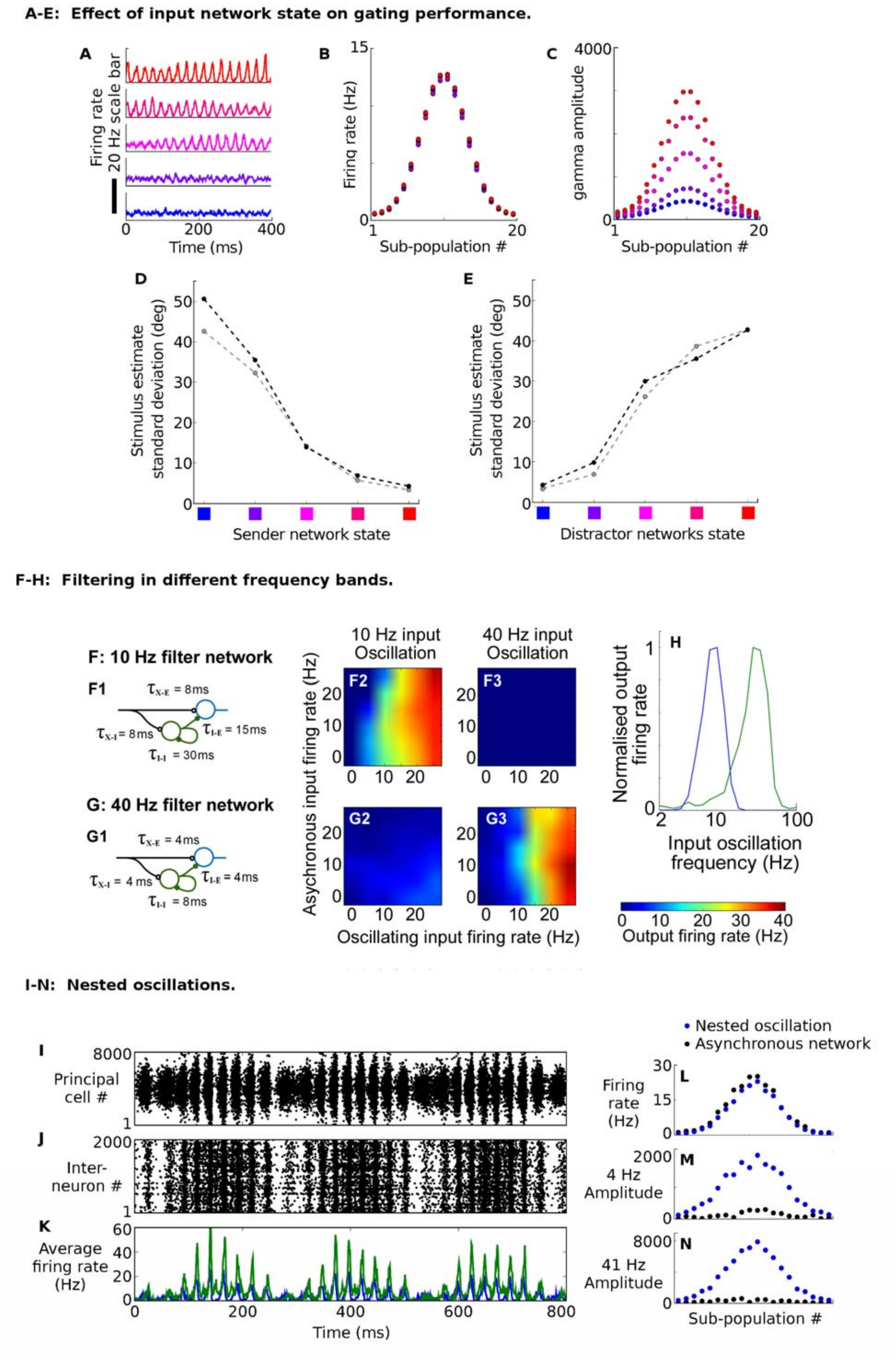

Figure S3 (related to Figure 6)

(A-E) Effect of Presynaptic Network State on Gating Performance

(**A**) Example traces showing network firing rate for 5 different states ranging from asynchronous to strongly oscillating. (**B**) Spatial pattern of neuronal firing rates in the 5 states, color-coded as in D. (**C**) Spatial pattern of gamma amplitude in the 5 states. (**D**) Effect of varying the state of the sender network on gating performance. Black trace shows standard deviation of stimulus estimate using filter network output, gray trace shows standard deviation of estimate from combined spike input. (**E**) Effect of varying state of distractor networks on gating performance.

(F-H) Filtering in Different Frequency Bands

Filtering networks with pass-bands at different frequencies were implemented by interneuron populations with different synaptic kinetics. **(F)** Filter network with passband at 10 Hz. (**H)** Filter network with passband at 40 Hz. (**F1**, **G1**) Diagram of filter networks showing the alpha function τ for each synaptic connection. (**F2**, **G2)** Filtering performance when driven by input oscillating at 10 Hz. The filter network was driven by a Poisson input consisting of an asynchronous component and a component sinusoidally modulated at 10 Hz. The firing rate of the filter network principal cells is plotted as a function of the firing rate of the oscillating and asynchronous components of the input. (**F3**, **G3)** Filtering performance when driven by 40 Hz input oscillation. (**H)** Pass-bands of the two filter networks. The filter networks were driven by a sinusoidally modulated input (Average firing rate per afferent 30 Hz, modulation depth 0.5) whose modulation frequency was varied between 2 and 100 Hz (Blue – 10 Hz filter network, green 40 Hz filter network).

(I-N) Nested Oscillations Create Amplitude Patterns in Multiple Frequency Bands

(**I-K**) Network activity during nested oscillations, a network oscillating at 40 Hz was modulated at 4 Hz by an externally applied sinusoidal current. (**I**) Principal cell spike raster. (**J**) Interneuron spike raster. (**K**) Average firing rate in principal cell (blue) and interneuron (green) populations. (**L-N**) Spatial pattern of firing rate and firing rate oscillation amplitude in the network with nested oscillations (blue) and for comparison in an asynchronous network (black). (**L**) Neuronal firing rate evaluated over 3 cycles of 4 Hz oscillation (750 ms). (**M**) Firing rate oscillation amplitude at 4 Hz. (**N**) Firing rate oscillation amplitude at 41 Hz. Amplitudes were calculated for Hanning-windowed sections of activity lasting 3 cycles of the oscillation frequency being measured (75 ms at 41 Hz, 750 ms at 4 Hz).

Supplemental References

Brunel, N., and Hakim, V. (2008). Sparsely synchronized neuronal oscillations. Chaos. *18*, 015113.

Chu, Z., Galarreta, M., and Hestrin, S. (2003). Synaptic interactions of late-spiking neocortical neurons in layer 1. Journal of Neuroscience *23*, 96.

Jonas, P., Bischofberger, J., Fricker, D., and Miles, R. (2004). Interneuron Diversity series: Fast in, fast out - temporal and spatial signal processing in hippocampal interneurons. Trends in Neurosciences 27, 30-40.

Marr, D., and Poggio, T. (1976). From understanding computation to understanding neural circuitry. AIM-357.
